# Environmental profiling of endocrine disrupting chemicals in ground water sources: an African perspective

**DOI:** 10.1039/d5ra04114g

**Published:** 2025-07-28

**Authors:** Esther A. Nnamani, Ajibola A. Bayode, Oluwaferanmi B. Otitoju, Moses O. Alfred, Martins O. Omorogie

**Affiliations:** a Environmental Science and Technology Research Unit, African Centre of Excellence for Water and Environmental Research (ACEWATER), Redeemer's University Ede PMB 230 Nigeria omorogiem@run.edu.ng dromorogiemoon@gmail.com; b Department of Chemical Sciences, Redeemer's University Ede PMB 230 Nigeria; c Institute of Chemistry, University of Potsdam Potsdam D-14476 Germany; d Chair of Urban Water Systems Engineering, School of Engineering and Design, Technical University of Munich Am Coulombwall 3 Garching D-85748 Germany mo.omorogie@tum.de

## Abstract

Emerging evidence substantiates that African groundwater is contaminated by a mixture of endocrine-disrupting compounds (EDCs). Groundwater pollution due to EDCs is a serious public health concern, particularly in regions with limited water resource management. To amplify this growing concern, the number of studies on EDCs in groundwater is significantly less than that on surface or wastewater discharge (influent and effluent). A systematic search of the major indexed databases was employed in extracting relevant literature for this study. The review discussed the state of the art of EDCs in African groundwater regarding their occurrence, sources, environmental fate, environmental health, and efficacy of predominant treatment technologies like adsorption and photocatalysis, as well as their drawbacks. Our analysis of the dataset covering multiple countries and years reveals frequent detections of pesticides, phenolics, steroid hormones, parabens, and phthalates. In many cases, detected concentrations in groundwater systems exceed international safety benchmarks up to mg L^−1^ in some locations. These exceedances, along with detections of unregulated or banned EDCs such as bisphenol A and some organochlorine pesticides, may imply potential human and ecological risks. Additionally, the data reveals spatial patterns: shallow urban wells and low-cost rural areas tend to have higher contamination, reflecting local sanitation and land-use influences. This study also reveals the widespread contamination of EDCs in the African groundwater systems and the dearth of data in sustainable treatment plans. Consequently, there is a need to navigate research focus on both the environmental profiling and treatment/remediation in this pivotal source of drinking water supply on the continent.

## Abstract 2

Emerging evidence substantiates that African groundwater is contaminated by a mixture of endocrine-disrupting compounds (EDCs).

## Graphical abstract

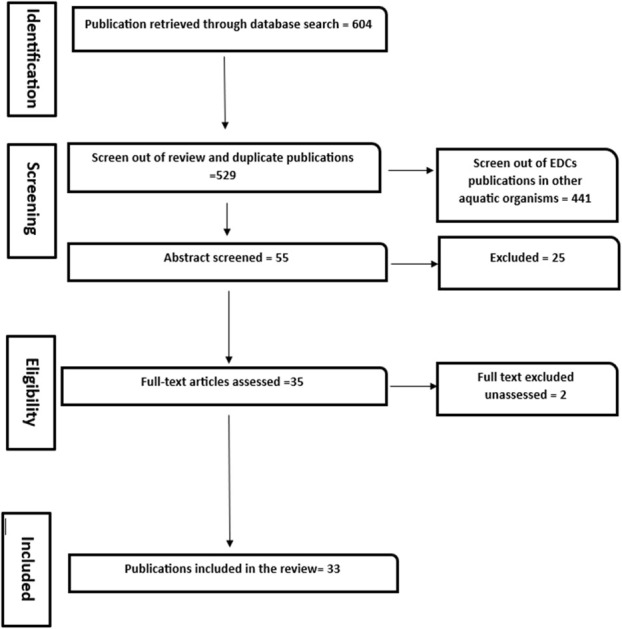

## 1. Introduction

The security of our drinking water supply is a public concern because drinking water acts as a potent exposure pathway for emerging contaminants such as endocrine-disrupting chemicals (EDCs) to bioaccumulate in humans.^1,2^ EDCs are emerging trace organic contaminants that are of grave concern to the well-being of the ecosystem. EDCs were first discovered in 1965, and they became a public health threat in the mid-70s.^3,4^ The term endocrine disruptor was first introduced by Colborn in 1991.^5^ According to the International Program on Chemical Safety (IPCS) of the United Nations (UN) and the World Health Organization (WHO) 2002, EDCs known as probable chemical disruptors are defined as complex exogenous substances or mixtures that have the tendencies or chemical properties to alter the normal functioning of the endocrine system in an organism, progeny, or sub-population.^6^ Global monitoring efforts have identified a wide array of EDCs in groundwater.^7–11^ However, recent global assessments by the Organization for Economic and Co-operate Development (OECD) reveal a pervasive yet unequal data and knowledge base regarding EDCs in groundwater. Also, the reports state that only 5% of the detected EDCs are actively monitored, highlighting the significant negligence in the environmental surveillance of EDCs in groundwater.^12^ Although EDCs seem to have received considerable interest, especially in developed countries, there is still limited knowledge on their environmental and human health effect, especially on the shores of Africa. Phthalates, parabens, pesticides, perfluorinated compounds, microplastics, heavy metals, polycyclic aromatic hydrocarbons, brominated flame retardants, and phenolic compounds represent this class of contaminants.^13^ They are used to produce a wide array of essential materials that are integral to our daily lives. EDCs are exogenous. Hence, they can alter the proper functioning of both human and aquatic organisms' endocrine systems and trigger serious reproductive and neurological disorders.^14^ Studies have evaluated the health risks associated with ingesting EDCs from contaminated groundwater sources, and some of the EDCs exceeded the tolerable daily limit stipulated by both the national and international regulatory bodies. For instance, organochlorine pesticides (OCPs) levels were monitored in some Nigerian groundwater samples. Heptachlor and methoxychlor exceeded the maximum residue limit established by the European Union, and the carcinogenic assessment showed that children were more vulnerable than adults.^15^ In South Africa, studies have confirmed the occurrence of BPA, phthalates, and estrogenic compounds in municipal groundwater with concentrations significantly high enough to raise health concerns.^16,17^ More so, EDCs exposure is associated with an increase in cases of non-communicable diseases such as cancer and diabetes.^18^ Recent studies have shown that EDCs can negatively impact insulin production and can be a potential cause of type 2 diabetes in humans.^19^ Common sources of EDCs include plastic ware, plasticizers, lubricants, personal care products, and pharmaceuticals. Table 1 shows frequently used EDCs in our day-to-day activities, along with their corresponding products and associated health risks.

**Table 1:** Overview of frequently detected endocrine-disrupting compounds in the African groundwater system

EDCs	Examples	Chemical structure	Product present in	Human health effects	References
Phthalate	Dimethyl phthalate	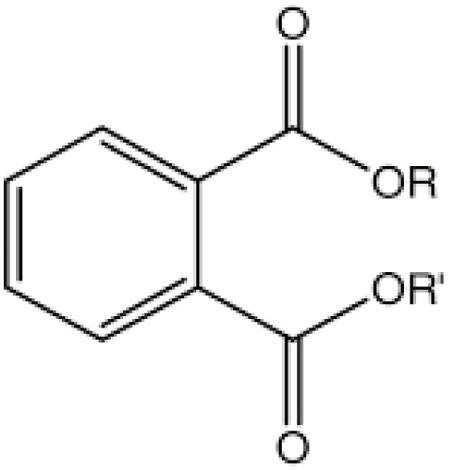	Food storage ware, baby feeding bottles, cables, and wires	Obesity in children and reduced maternal levels of thyroid hormones	53
Phenol	Bisphenol A	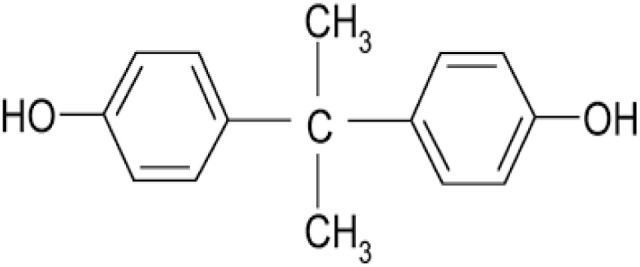	Water and food cans, water supply pipes, epoxy resin, polycarbonate resins	Skin and respiratory irritation, fertility disorder	52
Paraben	Methyl, ethyl, propyl, butyl parabens	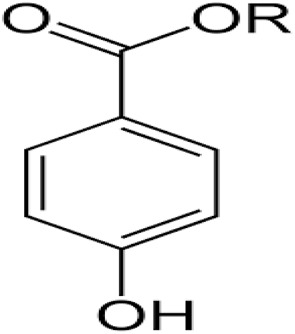	Deodorant, body creams, detergents, eye ointments	Carcinogenesis, hypersensitivity, and obesity	54
Pesticides	Organochlorine	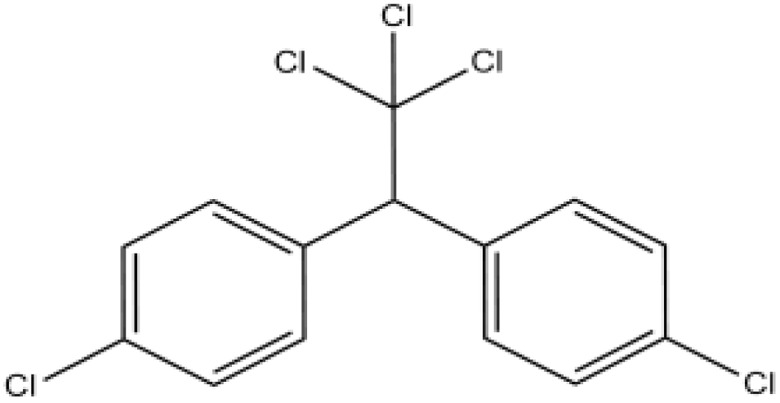	Pesticides, herbicides, plastics, polymers, and pharmaceuticals	Breast, prostate, stomach, and lung cancer	49
Polycyclic aromatic hydrocarbon (PAHs)	Naphthalene	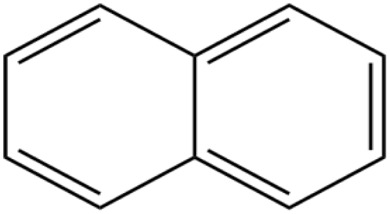	Synthetic dyes and drugs, coal tar, polymer production	Lung, skin, bladder, and gastrointestinal cancer	55
Pharmaceuticals	Antibiotics	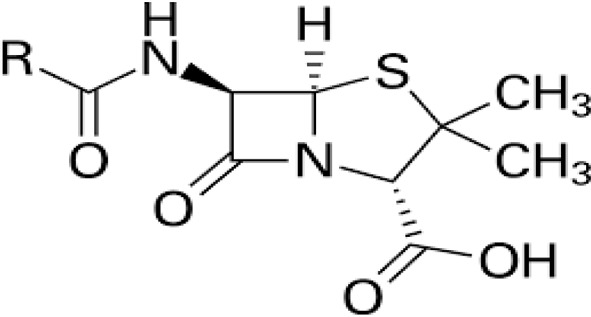	Treatment or prevention of diseases	Development of antibiotic-resistant genes or strains, liver and kidney toxicity, carcinogenic and mutagenic risk	56
Flame retardants (FRs)	Polybrominated diphenyl ethers (PBDEs)	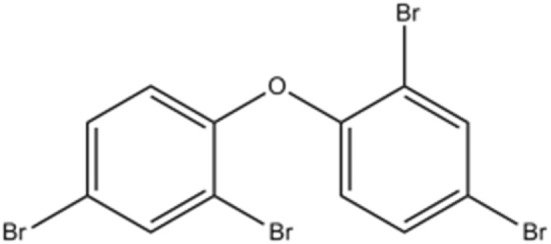	Automobile interior, electronic, plastics, and textile industries	Reproductive and neurological disorders for the fetus	57
Steroid hormones (SHs)	Estrogen, testosterone, progesterone	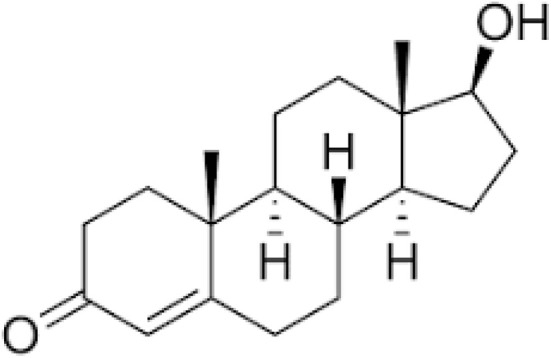	Vitamin D, glucocorticoids	Reproductive disorders, breast, prostate, and uterine cancer	58

Groundwater is the most reliable and continuous source of freshwater on Earth.^20^ It plays a crucial role in regulating the inflow and outflow of rivers and lakes and is essential for groundwater systems such as boreholes and wells. Groundwater is considered one of the primary and safest sources of freshwater for human survival.^21^

Unfortunately, the presence of certain EDCs in groundwater systems is alarmingly frequent and should be a significant concern. It is estimated that over 75% of the African population relies on groundwater as their main source of drinking water.^22^ Unlike other freshwater resources, the contamination of groundwater by EDCs is not well understood.^23^ Furthermore, the breakdown of EDCs in groundwater occurs very slowly due to unfavorable physical, chemical, and biological conditions that hinder the degradation processes in aquifers.^24^

Unfortunately, once EDCs find their way into groundwater, they may persist and can be transported over very long distances.^25^ Since some of the EDCs, especially pesticides and pharmaceuticals, have been detected, their correlation to adverse human health disorders *via* ingestion, inhalation, and dermal absorption is worthy of study. Researchers have revealed the relationship between the endocrine system and how organisms adapt to the environment. Consequently, any disruption in hormonal signals, metabolic reactions, and hormone reactions on receptors may induce physiological disorders in the human body.

Generally, the human health effects and environmental challenges associated with EDCs are sometimes difficult to manage globally, even in developed nations. However, in developing nations, especially the shores of Africa, EDCs seem to be poorly managed or overlooked in most cases, as evident in the dearth of data in many countries. There is a need for a comprehensive research study on the profiling of EDCs in groundwater resources, as it will be pivotal in developing a robust risk assessment framework and promulgating appropriate laws and regulations regarding the environmental management of our groundwater resources. EDC concentrations in groundwater are always detected, ranging from nanograms per litre (ng L^−1^) to micrograms per liter (μg L^−1^) to milligrams per liters (mg L^−1^) in extreme cases. EDCs are persistent, recalcitrant, and bioaccumulative. They are also lipophilic; therefore, they can be in human adipose tissue over time. The most frequently studied EDCs in African groundwater sources include phenolic compounds, phthalate esters, pharmaceuticals, and pesticides.^2,26^ Commonly used instrumentation technique for quantitative and qualitative analysis includes high-performance liquid chromatography and gas chromatography coupled with a mass spectrometer, ultraviolet, and flame ionization detectors.

The pervasive nature of EDCs in our groundwater requires urgent attention so that adverse health effects posed by these toxic organic contaminants can be eradicated or reduced to the bare minimum.^27^ Additionally, their complex chemical structure enables them to bypass conventional water treatment processes.^28^ Consequently, new remediation strategies are being deployed to curtail this severe public health challenge.^29^ Adsorption is one of the oldest forms of removing EDCs from groundwater resources. Different adsorbent materials have been developed and used. Adsorption has some deficiencies, and that is being addressed by more recent remediation technologies such as photocatalysis, bioremediation, oxidation, and membrane technology. Some researchers have reported the occurrence of EDCs in other aquatic systems such as surface water, estuarine water, wastewater influent, and effluent. For instance, the presence of PFAS in the African environment,^30–33^ pharmaceutical and personal care products in wastewater and surface water in Africa,^34–38^ Antibiotics in Africa,^39–41^ Emerging contaminants in the African aquatic environment,^42–45^ Microplastics in Africa,^22,46–48^ Organochlorine in environmental matrices in Africa^49,50^ and bisphenol A in Africa have been documented.^51,52^

However, there is minimal information on the occurrence of EDCs in African groundwater systems. Furthermore, the regulatory framework of EDCs is still in its infancy in many African countries. There is a need for the comprehensive geographical, seasonal, and spatial profiling of EDCs pollution in groundwater systems on the continent. Consequently, this review paper aims to provide African content regarding the environmental monitoring of EDCs in the continent, probable sources and environmental fate of EDCs, adverse health effects caused by these recalcitrant pollutants, and predominant remediation strategies with their advantages and limitations.

This review adopted the systematic approach to analyzing publications related to EDCs in African groundwater sources. This study will bring into the limelight the current realities of EDCs in African groundwater resources, as it is largely understudied. It will also help the continent to prioritize research studies in this regard and contribute to addressing the Sustainable Development Goals, particularly Goals 3.9 and 6.0, which are hinged on the reduction of deaths and illnesses from hazardous chemicals and contamination and the achievement of clean water and sanitation by the year 2030.

## 2. Review methodology

### 2.1 Study design

This review followed a well-detailed and structured Preferred Reporting Items for Systematic Reviews and Meta-Analyses (PRISMA) approach to evaluate investigative studies on the environmental occurrence of EDCs in African groundwater systems.

### 2.2 Data search strategy

Relevant literature using appropriate keywords such as “endocrine-disrupting compounds in African groundwater”, “groundwater contamination by EDCs”, “monitoring of EDCs in groundwater systems” and related terminologies were searched for across different scientific search engines and databases such as Science Direct, Google Scholar, Research gate, Scopus, Semantic Scholar, PubMed, African Journal Online (AJOL), and Web of Science. The search was for studies published from 2013 to 2024. A pool of 604 articles was retrieved. However, only 33 research articles were pertinent to the review scope.

### 2.3 Data inclusion and exclusion criteria

Relevant data and information were extracted from selected studies using a well-defined data inclusion and extraction format. Studies were included if they were conducted 2013 between 2024, focused on the occurrence of EDCs in African groundwater systems, were peer-reviewed studies, written in English, and provided sufficient quantitative data or research findings related to EDCs in African groundwater systems. For the exclusion criteria, studies on endocrine-disrupting metals were excluded. Studies on EDCs pollution in other aquatic matrices were removed. Duplicate and review articles lacking original research data were equally excluded. Also, inaccessible articles or articles with insufficient methodology were excluded. This approach ensured that only high-quality and relevant studies were finally considered for the systematic review.

### 2.4 Quality assessment

Following the PRISMA-established protocol, the reliability and quality of selected publications were scrutinized to ensure the inclusion of robust and valid data. The protocols included sample and study design, extraction and instrumentation techniques, and key findings from the study. Each study was carefully assessed. Any form of discrepancies was carefully reviewed before the inclusion. Endocrine-disrupting metals were excluded from this study.

### 2.5 Data analysis and synthesis

Extracted data was further scrutinized to provide a comprehensive overview of all the publications on the monitoring and geographical distribution of EDCs in African groundwater resources. The data were descriptively summarized, highlighting key findings on the concentration, probable source points, distribution, and potential health implications of the identified contaminants. The results were presented in a table format for clarity.

## 3. Results and discussion

### 3.1 Trends in environmental profiling of EDCs in African groundwater resources

**Fig. 1:**
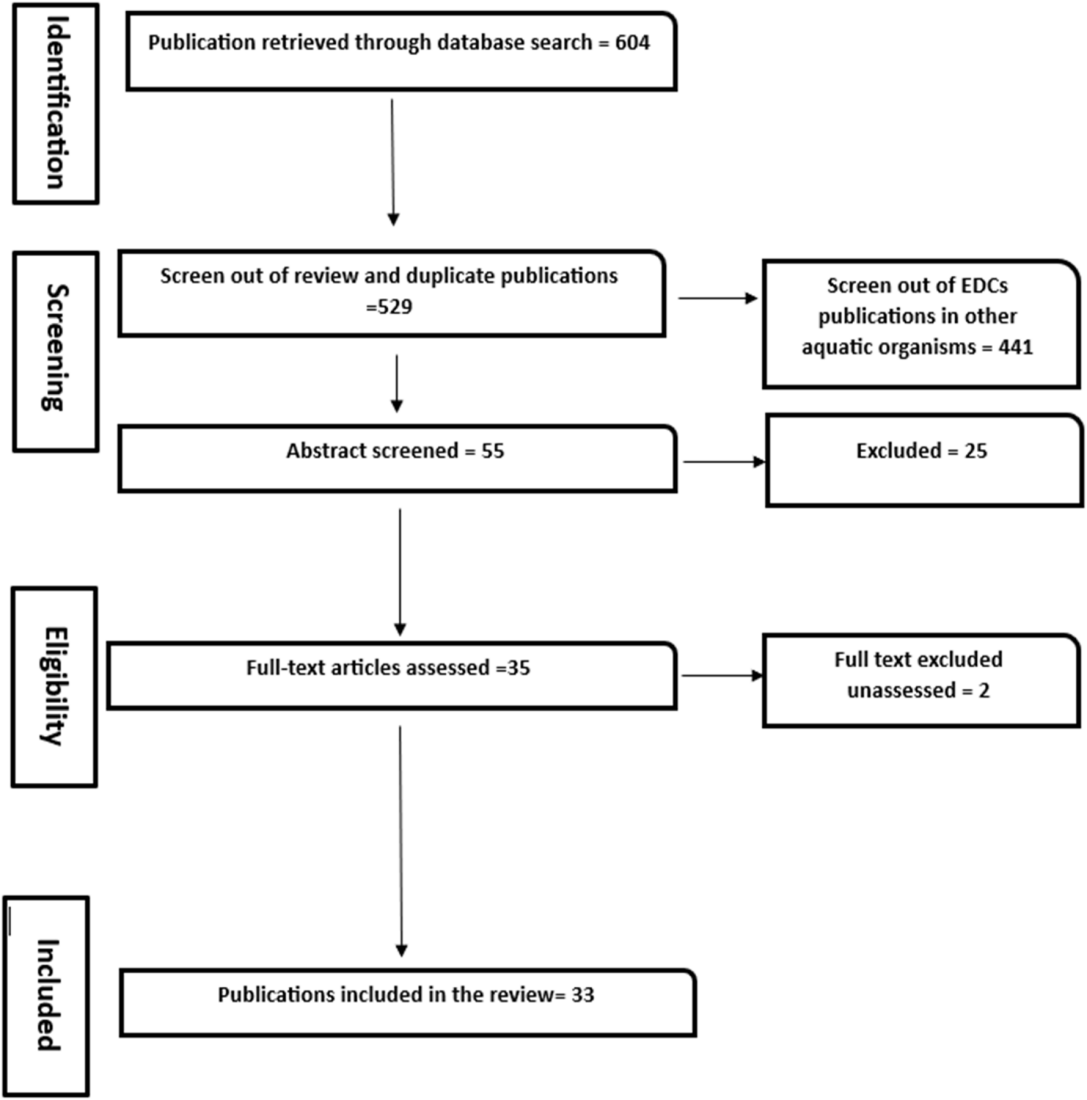
PRISMA diagram for retrieval of articles for the review study.

This study reviewed documented studies on the occurrence of EDCs in African groundwater, with the first study conducted in South Africa. Nigeria accounted for the highest number of publications, reflecting heightened concerns over EDC pollution in its groundwater systems. Most studies (2020–2024) detected EDCs such as BPA, pharmaceuticals, antibiotics, industrial solvents, pesticides, total hydrocarbons, parabens, and polybrominated diphenyl ethers (Table 3). While South African studies reported concentrations within regulatory limits, elevated EDC levels in Kenya, Nigeria, and Egypt warrant ecological and human health risk assessments. Notably, the highest EDC diversity was reported in Zambia (Fig. 1).

To assess the scope and variability of endocrine-disrupting compounds (EDCs) in African groundwater, a systematic review of peer-reviewed studies (2013–2024) was conducted. The compiled data encompassed multiple countries, including West Africa (Nigeria, Ghana), Northern Africa (Egypt, Tunisia), South Africa (the Republic of South Africa, Zambia), and East Africa (Kenya, Uganda). The studies from these countries focused on groundwater sources such as boreholes, hand-dug wells, springs, and tap water. Key parameters included EDC types, concentrations, detection frequencies, and analytical methodologies. The findings include over 40 distinct EDCs, with pesticides and their metabolites being frequently reported in rural and agricultural areas. Urban geo-political areas had pharmaceutical and personal care products being commonly reported. For example, atrazine, 2,4-D, and DDT were commonly detected in groundwater from Ghana, Nigeria, and Egypt, often from rural wells, whereas bisphenol A, triclosan, and parabens were mostly found in urban groundwater (South African and Kenyan wells). Shallow wells and springs typically had higher contamination than deep boreholes; a study in Zambia validated this claim. Seasonal sampling (wet *versus* dry) showed that many mobile EDCs such as. DEET, a sunscreen insect repellent, spiked after rains, affirming rapid infiltration into the groundwater system. The study revealed certain patterns; for instance, Nigerian groundwater samples, especially studies from 2018–2022, had the highest concentration of atrazine and phenolic compounds. Southern Africa had a mixture of pesticides, pharmaceuticals, and personal care products. North African data were sparser, but Egyptian wells near agricultural land also yielded detectable atrazine and DDT. The variety of EDCs detected from banned organochlorines to current-use herbicides underscores the diverse contamination sources. These findings are consistent with regional reviews noting that pharmaceuticals, plasticizers, personal care products, and pesticides have been found in African drinking water sources.

Methodologically, liquid–liquid extraction (LLE) followed by column clean-up was the predominant sample preparation technique. LLE involves the use of large organic solvents to concentrate analytes from water, but LLE suffers from inadequate or incomplete recovery and large usage of solvents. More recent studies reported the use of the solid-phase extraction (SPE) technique. Detection was done primarily by GC-MS for volatile and semi-volatile organics or HPLC–UV for non-volatiles. Instruments also differ in reported accuracy; most studies validated methods with recoveries of 70–120%, but inter-lab variability means that some low-level detections or non-detections should be interpreted cautiously. Nonetheless, contaminants were detected across all groundwater sources, underscoring the pervasive nature of EDC pollution. To guide science-based monitoring and policy actions, this study compiled prevalent EDCs in African groundwater sources with their current regulatory status. Table 2 shows a summary of identified priority EDCs in African groundwater. The compiled data emphasizes the urgent need for expanded monitoring and standardized regulatory frameworks to mitigate risks across the continent.

**Table 2:** Priority EDCs in African groundwater systems

EDC	Occurrence in African groundwater	Endocrine potency	Regulatory status	Reference
Bisphenol A	Detected in South Africa, Nigeria	Mimic female hormone, linked to reproductive, neurological, and metabolic disorders such as diabetes	Yet to be regulated on the continent, but some legislative actions are currently under review in South Africa	59 and 60
Phthalates	Detected in Nigeria	As a known endocrine disruptor, phthalates affect the reproductive system and trigger metabolic disorders	Not currently regulated, although it has been included in recent water surveys	7, 61
Phenolic compounds (nonylphenol, octylphenol, dehydrobenzene)	Frequently found in Nigerian, Kenyan, and South African groundwater sources	Estrogenic activity on both aquatic and human endocrine glands	Not yet regulated in Africa	62–65
Organochlorine pesticides (OCPs)	Levels of OCPs in groundwater in Nigeria and Kenya	Carcinogenic, neurotoxic, and alter the normal functioning of the endocrine glands	Partially regulated	15, 50 and 66
Pharmaceuticals and personal care products	Levels have been detected in many African nations, such as Egypt, South Africa, Nigeria	Disrupt the hormone and immune system, and contribute to antibiotic resistance in aquatic organisms	Not yet regulated	54 and 67–69
PFAS (PFOS, PFOA)	Although at very trace concentrations, it has been detected in Burkina Faso, Ivory Coast, Ghana, and Uganda groundwater systems	Persistently bio-accumulate in both human and aquatic organisms	Not yet regulated	70
Polycyclic aromatic hydrocarbons (PAH)	Elevated concentrations of PAHs have been detected in Nigeria's groundwater systems	Tendencies of disrupting thyroid hormones. Trigger anti-estrogenic effects that may impair reproductive health	The continent lacks harmonized regulations, and in some areas, regulatory measures are completely absent	55 and 71

### 3.2 Sources and environmental fate of EDCs in African groundwater sources

The environmental fate of EDCs is largely influenced by water currents and factors such as pH, temperature, conductivity, dissolved oxygen, and porosity.^72^ While some EDCs do not bioaccumulate due to their short lifespan, others have been reported to be recalcitrant, persistent in the environment, with a long shelf life. Adsorption and biodegradation are essential factors and processes that control the environmental fate (persistence, mobility, and toxicity) of EDCs. Adsorption influences the partition ratio of EDCs in the solid–liquid phase, while biodegradation can alter or destroy the structure and morphology of EDCs.^72^ The fate of most EDCs after permeating the aquifers may be determined by the octanol–water partition coefficient (log *K*_ow_) and the octanol–water distribution coefficient (log *D*_ow_). A high log *K*_ow_ indicates that an EDC is hydrophobic and will partition more in the organic phase like sediment, fat and oil, and will be insoluble in aquatic systems. Such EDCs tend to stay longer in the adipose tissues of organisms and potentially disrupt the endocrine system. EDCs with low *K*_ow_ are hydrophilic and dissolve easily in aqueous media, increasing their transboundary and leaching effects, thereby leading to their widespread distribution in water systems. Moderate *K*_ow_ EDCs adsorb onto organic matter, sediments, and dust particles. Their presence in the aquatic matrix may be minimal, however, they bioaccumulate and lead to long-term environmental persistence. The degradation and treatment process for EDCs are also influenced by their *K*_ow_ values. While high *K*_ow_ values may be largely removed through the adsorption process, EDCs with low *K*_ow_ may favour microbial degradation and advanced oxidation processes because of their affinity to the aquatic matrix. Other environmental criteria, such as unique characteristics of the EDCs, redox reaction, their loading capacity, and water residence time, are important in determining the fate and life of EDCs in the aquatic environment. There are many pathways to groundwater contamination, with landfill being the highest and agricultural surface run-off the least.^4^

### 3.3 Prevalent factors for EDCs distribution in African groundwater sources

**Fig. 2:**
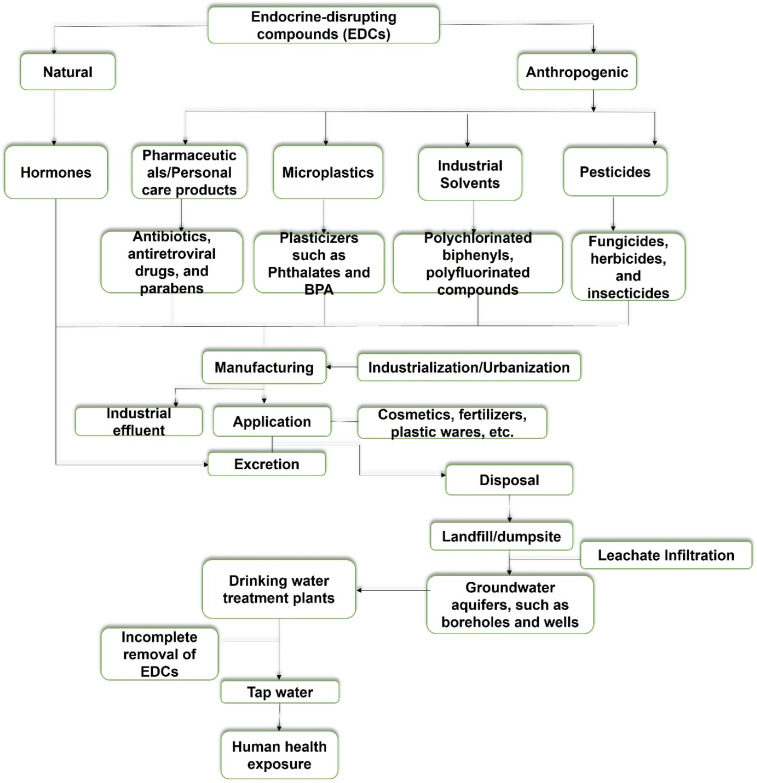
EDCs contamination pathways into groundwater systems.

Factors governing the distribution of EDCs in African groundwater resources are influenced by the continent's unique socio-economic, environmental, and infrastructural facilities.^73^ For instance, the region is known largely for its vast land space used for agricultural activities. As a result, there is a high usage of unregulated pesticides such as bactericides, fungicides, herbicides, and fertilizers.^74^ The region also relies on irrigation systems, especially during the dry seasons. This practice over an extended time can reduce our water quality, especially our groundwater sources like hand-dug wells and boreholes.^74^ These compounds, when applied to farmland, accumulate in the upper soil layers. During rainfall events or through irrigation, especially in regions that are reliant on groundwater for crop production, they are leached into subsurface aquifers. This transport is facilitated in regions with highly permeable soil, such as sandy or loamy soils, which permit rapid infiltration of water and solutes. Rapid urbanization and industrialization have worsened land pollution, as some homes in the region have dumpsites not too far from their settlement.^75^ Additionally, the discharge of poorly treated waste from chemical, pharmaceutical, and mining plants is one of the essential modes of EDCs distribution in groundwater resources. The hydrogeological characteristics of most African groundwater systems, especially in the sub-Saharan region, are shallow with fragile wetlands and coastal aquifers.^76^ Therefore, they are prone to leachate infiltration from the identified point sources. The arid and semi-arid areas of the region have limited groundwater systems, leading to increased concentration of EDCs due to a low dilution factor. The geological texture of our soil type, which is sandy and porous in some areas of the region, also facilitates rapid infiltration of EDCs into our groundwater systems. Clay-based soil may slow EDCs transport; however, it encourages localized accumulation.^77^ Prevalent cultural and behavioral practices, such as localized production of personal care products in homes, contribute significantly to the growing presence of EDCs in domestic wastewater and consequently groundwater systems on the continent, especially as typified in some of the studies in Nigeria. Pharmaceuticals such as anti-retroviral drugs were commonly reported in Southern Africa. Additionally, the weak regulatory framework, unregulated waste disposal, limited wastewater treatment plants, and soil topography worsen EDCs pollution in groundwater on the shores of Africa.^78^ The environmental transport of EDCs into groundwater resources is summarized in Fig. 2.

### 3.4 Methods for profiling, sample preparation, and instrumentation techniques of EDCs

Profiling of endocrine-disrupting compounds (EDCs) in groundwater necessitates meticulous planning and the use of appropriate sampling strategies to ensure accurate representation of their occurrence. Among the most employed techniques is single-grab sampling, which involves the collection of a single water sample at a specific location and time. This approach offers simplicity and cost-effectiveness, but cannot capture temporal variations in EDC concentrations.^79^ Composite-grab sampling provides an improvement by integrating multiple samples from different points within the same site. However, a more comprehensive characterization of EDCs in groundwater can be achieved by employing advanced approaches such as passive sampling, time-weighted composite sampling, high-resolution mass spectrometry (HRMS), and isotope tracer techniques in conjunction with traditional grab sampling.^80^ These methodologies enhance temporal and spatial resolution, offering more representative profiles of EDC contamination.^80^ The heterogeneity and broad classification of EDCs pose significant analytical challenges, particularly in their profiling, sample preparation, and detection in complex groundwater matrices.^81^

Effective sample preparation is crucial for the successful instrumental analysis of EDCs. Several extraction techniques have been utilized in studies across Africa, with the most notable being liquid–liquid extraction (LLE), solid-phase extraction (SPE), soxhlet extraction, and microwave-assisted extraction (MAE). LLE, one of the earliest techniques in environmental analysis, operates on the principle of partitioning analytes between two immiscible liquid phases based on solubility differences. Solvents such as acetone and dichloromethane have been reported to enhance partitioning efficiency. Although LLE has been employed to extract pesticides and phenolic compounds, its utility is limited when dealing with complex mixtures of EDCs. This limitation has led to a shift toward more efficient alternatives.^82^ Solid-phase extraction (SPE) has emerged as the most widely adopted technique in African groundwater studies, as shown in Table 3. SPE is advantageous due to its reduced solvent consumption, shorter processing times, and greater analytical precision. The method involves passing water samples through cartridges packed with selective adsorbents, which retain target analytes until elution with a suitable solvent. SPE also allows multi-class extraction, facilitated by the use of versatile cartridges tailored to the physicochemical properties, such as polarity, of different EDCs.^83,84^ Other innovative techniques, including microwave-assisted extraction (MAE) and solid-phase microextraction (SPME), are increasingly favored for their operational efficiency and capacity to handle trace-level contaminants in complex matrices.^85^

**Table 3:** Comparative analysis of edcs studied in african groundwater sources^a^

Country & year of study	EDCs	Location	Groundwater source	Instrumentation techniques	Remark	Regulatory observations (WHO, USEPA, and EU)	Ref.
Nigeria, 2014	Total petroleum hydrocarbon (TPH)	Rural	Borehole	LLE, GC-FID	Elevated concentration of TPH was detected in the studied sites. As the depth increased, the concentration of TPH decreased	TPH is not directly regulated by the WHO, USEPA, or EU	93
Nigeria, 2019	10, chlorinated, nitrogen-containing, and alkyl phenolic compounds	Rural	Hand-dug wells	SPE/GC-FID	The phenolic compounds, excluding 2-chlorophenol, were detected in the sites studied. The concentration of phenolic compounds ranged from ND to 0.0904 ppm. Nonylphenol (NP), 2,4-dinitrophenol, and 2,4,6-trichlorophenol (TCP) were the prevalent EDCs across the sampled sites	NP: EU EQS = 0.3 μg L^−1^; TCP: EPA MCL = 70 μg L^−1^; DNP: USEPA screening level ≈ 70 μg L^−1^. Measured values exceeded several guideline limits	6
Nigeria, 2022	Organochlorine pesticides (OCPs)	Semi-urban	Shallow wells and boreholes	LLE/GC-ECD	Elevated concentrations of DDT, heptachlor, and methoxychlor in comparison with other studied OCPs	Heptachlor and methoxychlor exceeded the EU MRL of 0.00002 ng L^−1^	15
Nigeria, 2022	Bisphenol A	Urban	Hand-dug wells	NS	BPA was detected in the samples ranging from 0.63–0.68 μg L^−1^	Reported values exceed the WHO and USEPA (0.1 μg L^−1^) reference dose	51
Nigeria, 2023	Bisphenol, nonylphenol, and octylphenol (OP)	Rural and semi-urban areas	Springs, hand-dug wells, and boreholes	LLE/GC-MS	BPA and OP were not detected, but NP was detected at a very low concentration	NP values were below the EU EQS	60
Nigeria, 2023	2,4-Dinitrophenol (2,4-DNP), phenol (PHE), and 2,4,6-trichlorophenol (2,4,6-TCP)	Rural and urban	Hand-dug wells and boreholes	SPE/HPLC-UV	Phenol had the highest detection frequency. Higher concentrations of the targeted EDCs were more in GW than in SW	Reported values were significantly high and exceeded the USEPA MCL of 70 μg L^−1^ for 2,4,6-TC & screening level of ≈ 70 μg L^−1^ for 2,4-DNP	62
Nigeria, 2024	Polybrominated diphenyl ethers (PBDEs)	Semi-urban and urban	Hand-dug well	LLE/GC-MS	PBDEs were detected at elevated concentrations in most of the studied sites	PBDE values exceeded the EU EQS of 0.0005 μg L^−1^ by several order of magnitude	94
Nigeria, 2024	Catechol and hydroquinone	Rural and urban	Hand-dug wells and boreholes	SPE/HPLC-UV	Catechol had a higher detection frequency than hydroquinone	Both compounds are yet to be regulated. However, hydroquinone has been categorized as hazardous in occupational settings	95
Nigeria, 2024	Ampicillin, chloramphenicol, ciprofloxacin, metronidazole, tetracycline, methylparaben, ethylparaben, propylparaben, and butylparaben)	Rural and urban	Hand-dug wells and boreholes	SPE/HPLC-UV	The mean concentrations of methylparaben and ciprofloxacin were higher in GW than in SW	No specific limits for pharmaceuticals in drinking water, but some of them, like ciprofloxacin, are under environmental monitoring. Parabens are yet to be regulated in drinking water by the main regulatory bodies	67
Nigeria, 2024	Methylparaben, ethylparaben, propylparaben, butylparaben ampicillin, chloramphenicol, ciprofloxacin and metronidazole	Rural	Hand-dug wells	LC-UV	EDCs GW concentrations were as high as 7846 (MET), 1137 (CIP), 342 (MeP), 295 (EtP), 299 (PrP), and 400 μg L^−1^ (BuP). Values from this study were high in comparison with other reported studies	Parabens are yet to be regulated in drinking water. Also, there are no benchmark values for pharmaceuticals	68
Ghana, 2024	Bisphenol A, chloramphenicol, 17-alpha ethynyl estradiol, 17-beta-estradiol, and estrone	Urban	Tap water (borehole)	SPE/HPLC-UV	Estrone, followed by 4-nonylphenol, was detected at elevated concentrations across all sampled sites	BPA exceeded the WHO (0.1 μg L^−1^) and EU EQS (0.3 μg L^−1^) limits. Estrone, 17-α ethynyl estradiol, and 17-β estradiol exceeded EU watch List thresholds (0.000035–0.0035 μg L^−1^), indicating high ecological risk. Chloramphenicol is not regulated in drinking water but is banned in veterinary use due to its toxicity	96
Tunisia, 2013	Carbamazepine	Urban	Well	Line SPE/LC–ESI-MS/MS system	The concentration of the targeted analyte ranged from 20.4 to 910 ng L^−1^. Although carbamazepine was not detected in some samples	Not currently regulated in drinking water by the WHO, USEPA, or EU. However, it is included on the EU watch list for monitoring due to persistence and ecotoxicity	92
Egypt, 2020	BPA, methylparaben (MeP), ethylparaben (EtP), propylparaben (PrP), butylparaben (BuP), and *o*-phenylphenol	Rural/urban	Groundwater	UPLC-MS/MS	This study reported the highest levels of MeP globally as at when it was carried out. MeP (16.3%) was the most frequently detected EDC in DW (GW), followed by BPA (14.5%), PrP (6.9%), and BuP (6.2%)	BPA values were several magnitudes higher than the WHO (0.1 μg L^−1^) and EU EQS (0.3 μg L^−1^) limits. Parabens (MeP, EtP, PrP, BuP) and *o*-phenylphenol are not yet regulated in drinking water by the WHO, USEPA, or EU	69
Zambia 2015	Over 1000 EDCs including caffeine, dieldrin, *N*,*N*-diethyl-*m*-toluamide, 1,3-dichlorobenzene, atrazine, 4,4-DDT, beta-BHC (beta-HCH), triacetin, triclosan, and *o*,*p*′-DDT	Peri-urban, industrial land use	Shallow wells and boreholes	Double LLE/multi-residue GC-MS method	*N*,*N*-Diethyl-*m*-toluamide (DEET) was the most prevalent across all samples, with about 85% detection frequency	The most prevalent compound (DEET) in the study is yet to be regulated. Reported values of atrazine were below the permissible limit of 100 μg L^−1^ and 3 μg L^−1^ for WHO and USEPA, respectively. Several of the studied compounds lack formal drinking water guidelines	97
South Africa, 2024	4-Chlorophenol and 2,4-dichlorophenol	Urban	Tap water	SPE/HPLC-DAD	EDCs were detected in tap water. However, the measured levels are below the stipulated limits in South Africa	Targeted EDCs are not currently regulated in drinking water by the WHO or the USEPA. Occurrence levels in the study remain below taste threshold guidelines and below the EU/UK environmental standards for protection of aquatic ecosystems	98
Uganda, 2021	26 antibiotics, 20 hydrocarbons, including 16 polycyclic aromatic hydrocarbons (PAHs), and 59 pesticides	Urban	Shallow groundwater	SPE/GC-MS/MS and LC/MS	Ampicillin and benzylpenicillin were the most frequently detected antibiotics. Naphthalene, xylene, anthracene, and fluoranthene were frequently detected hydrocarbons. Cypermethrin and metalaxyl were the most detected pesticides in the study. Eight banned organochlorines (endosulfan and DDT) were also detected at low levels, although exceeding the stipulated limits by the EU water standard	Total pesticide concentrations exceeded the EU guideline. While pharmaceuticals are currently under watch. Total PAHs exceeded the WHO and EU limit of 0.10 μg L^−1^. OCPs DDT and endosulfan were higher than the WHO/EPA/EU: 0.0005–1 μg L^−1^; EU pesticide ≤0.1 μg L^−1^	99
Kenya, 2016	Antibiotic, anti(retro)viral, analgesic, anti-inflammatory and psychiatric drugs	Urban	Groundwater well	HPLC MAT95XP HRMS	Pharmaceuticals were detected at low concentrations in groundwater when compared with other aquatic matrices	Targeted analytes in the study are currently being monitored because of their resistance to chlorination and environmental persistence	100
Kenya, 2022	14 pharmaceuticals, 5 personal care products, and 9 pesticides	Rural	Groundwater wells in a river basin	SPE-UHPLC-HRM	Parabens (methylparaben) were the most dominant, followed by antiretroviral (nevirapine) drugs. Reported values were higher than developed nations	No established benchmark for the compounds, pharmaceuticals are currently being reviewed by the EU	91

a Note: SPE-solid phase extraction; LLE-liquid-liquid extraction; GC-FID-gas chromatography-flame ionization detector; GC-MS-Gas chromatography-mass spectrometry; HRMS-high-resolution mass spectrometry (HRMS); LC-LC-ESI-MS/MS: liquid chromatography-electron spray ionization tandem mass spectrometry; HPLC-UV-high-performance liquid chromatography-ultraviolet detector; UPLC-MS/MS-ultra-pure liquid chromatography-tandem mass spectrometry; ultra-high performance liquid chromatography (UHPLC)-high-resolution orbitrap mass spectrometry (HRM); GW-groundwater; SW-surface water; DW-drinking water; EQS-environmental quality standard; MRL-maximum residue limit.

The quantitative and qualitative assessment of EDCs in groundwater demands high sensitivity, accuracy, and selectivity due to the typically low concentrations (often in the parts-per-billion or parts-per-trillion range).^86^ Mass spectrometry (MS), particularly when coupled with separation techniques like gas chromatography (GC) or high-performance liquid chromatography (HPLC), plays a pivotal role in EDC detection.^87^ Recent advancements emphasize the use of tandem mass spectrometry (MS/MS) over traditional detectors such as diode array detectors (DAD) or ultraviolet detectors (UV), owing to its superior sensitivity and specificity.^88^ The integration of liquid chromatography with tandem mass spectrometry (LC-MS/MS) has become a preferred analytical platform for EDCs due to its robustness in handling multi-residue analysis and ultra-trace detection limits.^89,90^ While LC-MS/MS is sophisticated and resource-intensive, it provides unmatched analytical depth, enabling simultaneous identification and quantification of diverse EDCs. The accuracy of this instrumentation, however, is contingent upon thorough sample pretreatment, including extraction, cleanup, and pre-concentration, emphasizing the necessity of optimized sample preparation protocols in EDC pollution in groundwater studies.

### 3.5 Case studies of EDC contamination in African groundwater

Studies on the occurrence and distribution of endocrine-disrupting compounds (EDCs) in African groundwater remain limited, with significant gaps in most nations. However, available studies reveal both spatial and temporal variability in EDCs contamination across different regions on the continent. One of the earliest and most comprehensive environmental assessments of EDCs was conducted in Zambia by Sorensen *et al.* (2015). The study screened for 1000 compounds and detected 27 analytes, including *N*,*N*-diethyl-*m*-toluamide (DEET, the most frequent), triclosan, trihalomethanes, herbicides, and chlorinated compounds. Spatial analysis reveals that contamination was highest in shallow wells within low-income areas, where lifestyle patterns and socioeconomic conditions like inadequate sanitation enhanced EDC migration into groundwater. In contrast, boreholes in high-cost housing areas showed minimal contamination, with DEET concentrations five times lower and rarely coexisting with bromacil or trichloroethylene. Temporally, seasonal variations were notable, with DEET levels spiking during rainy seasons, suggesting leachate infiltration as a primary transport mechanism.

Similar spatial disparities emerged in South Africa, where phenolic compounds (4-chlorophenol and 2,4-dichlorophenol) in Cape Town's groundwater were below FDA and national regulatory limits, a finding attributed to the city's advanced wastewater treatment using membrane reactors. While no carcinogenic risks were identified, non-carcinogenic health risks persisted (43). In Nigeria, several studies have revealed an alarming profile of EDCs groundwater contamination, both in urban and rural areas. Compounds such as bisphenol A (BPA), nonylphenol (NP), octyl phenol (OP), parabens, phenolics, organochlorine pesticides, and antibiotics have been reported at elevated concentrations, often exceeding WHO or USEPA guideline values. For instance, parabens and antibiotics like metronidazole and ciprofloxacin were found at concentrations up to 7846 μg L^−1^, which are significantly above international safety thresholds. Studies from 2024 also report the presence of PBDEs, catechol, hydroquinone, and 2,4-dinitrophenol, indicating a broad spectrum of contamination in hand-dug wells, boreholes, and springs. Notably, phenol and 2,4,6-trichlorophenol frequently exceeded permissible limits, especially in rural groundwater systems with minimal protection.^54,62,63^ EDCs pollution in groundwater samples from urban boreholes in Ghana also revealed concerning levels of bisphenol A, estrone, and 4-nonylphenol, all of which exceeded WHO allowable limits of 0.1, 1.0, and 0.3 μg L^−1^ respectively. The endocrine-disruptive potential of these compounds underscores the urgent need for policy, surveillance, and remediation.

In North Africa, Egypt reported widespread EDC pollution in both rural and urban groundwater sources, with methylparaben (16.3%) and BPA (14.5%) emerging as the most frequently detected analytes. This pattern suggests sustained contamination, possibly linked to widespread use of personal care products and inadequate wastewater disposal over time.^69^ Tunisia recorded carbamazepine in well water at concentrations ranging from 20.4 to 910 ng L^−1^, with some temporal variations likely influenced by seasonal pharmaceutical discharge and hydrogeological factors. Though levels were below immediate risk thresholds, chronic low-dose exposure remains a concern. Across East Africa, Kenya and Uganda have demonstrated the presence of EDCs in groundwater. In Kenya, methylparaben, nevirapine, and carbamazepine were identified at relatively low concentrations; however, their detection still raises concern due to the vulnerability of infants and children.^91,92^ Uganda's 2021 study revealed 26 antibiotics, 20 hydrocarbons, and 59 pesticides in shallow urban groundwater. Alarmingly, banned organochlorine pesticides like DDT and endosulfan exceeded EU safety thresholds, reflecting both current use and historical persistence.

Over the past decade, the concentration and diversity of EDCs in African groundwater have shown a clear upward trend. Early studies (2013–2015), such as those in Tunisia and Zambia, primarily identified legacy pollutants like carbamazepine, DDT, and DEET at relatively low concentrations, typically in the ng L^−1^ to low μg L^−1^ range. As analytical methods improved and environmental monitoring expanded, subsequent studies (2016–2019) began detecting a broader array of compounds, including alkyl phenols, parabens, and pharmaceutical residues such as nevirapine and analgesics, particularly in groundwater from rural and semi-urban settings in Kenya and Nigeria. By 2020–2022, a marked increase in EDC concentrations was reported across multiple countries. For instance, Egypt documented the highest global levels of methylparaben (16.3% detection frequency), while Nigeria and Uganda reported elevated concentrations of organochlorine pesticides, antibiotics, and hydrocarbons. Most notably, between 2023 and 2024, several Nigerian studies recorded alarmingly high levels of pharmaceuticals and preservatives in hand-dug wells and boreholes, with methylparaben, ciprofloxacin, and metronidazole reaching concentrations of 342, 1137, and 7846 μg L^−1^, respectively. This temporal escalation reflects growing urbanization, increased use of personal care products and antibiotics, and insufficient waste disposal practices, coupled with improved detection sensitivity. In a similar trend, the spatial heterogeneity observed across African nations, ranging from highly contaminated rural and peri-urban zones to relatively less polluted high-income urban neighborhoods, highlights the influence of land use, socioeconomic factors, and infrastructure disparities on groundwater quality. In addition, temporally, seasonal fluctuations, especially during the rainy season, consistently lead to elevated EDC concentrations due to enhanced leachate movement and surface runoff. While specific maximum contaminant levels (MCLs) for many EDCs such as BPA, parabens, and antibiotics remain undefined by WHO or USEPA, existing toxicological evidence indicates their potential for endocrine disruption, reproductive toxicity, and carcinogenic effects. The recurring detection and the progressive rise in the concentration of EDCs in African groundwater highlights an urgent need for comprehensive regulatory frameworks, improved wastewater treatment, and routine groundwater surveillance to mitigate long-term ecological and public health risks across the continent. Table 3 presents a comparative overview of studies carried out on EDC pollution in African groundwater resource systems.

## 4. Regulation and environmental health impact of EDCs in groundwater

The eco-toxicity effect of EDCs can be very grave even at trace levels because of the impact on public health, benthic organisms, freshwater aquatic life, and wildlife.^72^ Structurally, EDCs are similar to several natural hormones. For instance, 17-α-ethinylestradiol, a synthesized estrogen used as a birth control pill, was found to cause feminization in a male fish after prolonged exposure at 5–6 ng L^−1^.^20^ Although EDCs have been detected in aquatic organisms around the continents, studies on their toxic effect are lacking. It has been scientifically validated that the continuous release of these contaminants into our groundwater resources is closely linked to the increased concentration of antibiotic-resistant genes and bacteria, ultimately affecting the efficiency of antibiotics.^40,41^ This poses a significant hazard to both humans and aquatic organisms, as it contributes to the development of drug resistance in pathogens responsible for severe diseases like malaria. Children are especially vulnerable. Exposure to EDCs has been linked to rising cases of childhood obesity, hormonal imbalances, and developmental disorders.

Regrettably, most developing nations, including Nigeria, do not have regional and national guidelines or regulatory frameworks on EDCs.^101^ Several existing chemical management systems focus on general hazardous substances without addressing the unique and persistent nature of endocrine disruptors. At the continental level, frameworks like the Bamako convention (1998), which prohibits the importation of hazardous waste into Africa, and international treaties such as the Stockholm, Basel, and Rotterdam Conventions, offer some level of control. Unfortunately, most of these policies are not EDC-specific. The first country to set a boundary for an EDC on the continent was South Africa in 2011, including restrictions on specific consumer products like infant feeding bottles.^77^ In contrast, Nigeria's National Environmental Standards and Regulatory Enforcement Agency (NESREA) is yet to develop a specialized protocol for EDCs monitoring and management due to a lack of technical knowledge, research infrastructure, funding, and support required for comprehensive EDCs studies. Other countries such as Kenya, Uganda, and Zimbabwe have participated in initiatives to reduce the release of hazardous chemicals; these efforts are often broader in scope and not specifically targeted at EDCs in water.^102^ Consequently, this deficiency in most developing countries has resulted in increased release of EDCs into the environment, including groundwater aquifers, with corresponding severe health effects on the well-being of the ecosystem. Several pollution monitoring campaigns revealed that exposure to EDCs may be higher in Africa and Asia compared to other continents, posing severe public health threats in these regions.^73,103^ For instance, one of the adverse health complications of EDCs exposure in children is obesity, and the number of children with abnormal body mass index is a growing concern in the region, as the number keeps growing geometrically. In a study carried out by Onyekachi *et al.* (2019), the estimated chronic daily intake of 2-nitrophenol, 2,4-dimethylphenol, 4-nitrophenol, 2-chlorophenol, and bisphenol A was below the stipulated oral reference doses. However, the risk quotients for nonylphenol and 2,4,6-trichlorophenol were greater than 1. Therefore, they have been identified as the major EDC contributors to the public health menace in the exposed communities. Predominantly, there are three exposure pathways of EDCs into human physiology, namely: ingestion, dermal absorption, and inhalation. Ingestion seems to be dominant of the three because of the direct consumption of water into our system.^69,104,105^

## 5. Perspectives on EDC mitigation strategies in African groundwater systems

Although several studies in Africa have investigated mitigation and treatment options for EDCs in wastewater treatment plants, there is a notable paucity of research focused specifically on the removal of EDCs from groundwater systems in Africa.^38^ Moreover, the limited effectiveness of conventional treatment methods and the prohibitive costs associated with advanced technologies, such as photocatalysis, electrochemical oxidation, nanotechnology-based systems, and membrane filtration, underscore the urgent need for low-cost, scalable solutions. This necessity calls for intensified research efforts by environmental and materials scientists in the region. Among the viable alternatives, adsorption has emerged as a cost-effective and efficient approach due to the high surface area and tailored surface chemistry of adsorbent materials, as well as their availability and potential to yield non-toxic byproducts. Eco-friendly adsorbents derived from agricultural and industrial waste, such as rice husks, kaolin clay, eggshells, palm kernel chaff, and plantain or banana peels, have shown great promise. These materials, often used in the synthesis of biochar or activated carbon, exhibit enhanced surface area, the presence of oxygen-containing functional groups, and high adsorption capacity, making them sustainable options for groundwater remediation.^106^ Also, the application of the activated carbon adsorption process has been used in the remediation of tetracycline and bisphenol A in groundwater. These methods significantly reduce the concentration of semiconductors required for the removal of EDCs in the aquatic matrix. This approach was widely used across all the reviewed studies in Africa. Typically, the adsorption process is divided into two main classes: physisorption (weak intermolecular forces) and chemisorption usually a covalent bond formation between the surface of the adsorbent and the absorbing material. Numerous studies have demonstrated that activated carbon (AC) exhibits a high capacity for removing a wide spectrum of representative endocrine-disrupting compounds (EDCs) from both synthetic and real wastewater across laboratory, pilot, and full-scale treatment systems. Research on EDC removal using AC primarily focuses on evaluating its removal efficiency across various water matrices, as well as examining the influence of EDC physicochemical properties and the characteristics of AC derived from different precursor materials. Largely, there are more laboratory-simulated experiments than industry or real-life applications. In studies conducted in Kenya, Shikuku *et al.* (2020) and Okello *et al.* (2017) demonstrated the effectiveness of sugarcane bagasse-derived biochar (CBG) for the removal of emerging pharmaceutical contaminants from aqueous systems. Specifically, sugarcane bagasse biochar achieved a 78% removal efficiency for sulfamethoxazole, with a reported maximum adsorption capacity of 128.8 mg g^−1^. Additionally, carbamazepine, another widely detected pharmaceutical compound, was successfully removed using sugarcane bagasse biochar magnetically modified with iron oxide (α-Fe_2_O_3_-CBG), achieving a removal efficiency of 60.9%.^107,108^ Several studies in Nigeria have explored the use of locally available materials for the adsorption of EDCs from aqueous environments. Tetracycline and bisphenol A, two frequently detected EDCs, were effectively removed using kaolin clay, demonstrating removal efficiencies of 84% and 51%, respectively. The maximum adsorption capacities recorded were 30 mg g^−1^ for tetracycline and 23 mg g^−1^ for bisphenol A, respectively.^109^

Photocatalysis stands out as a promising method for environmental remediation across the African continent, primarily due to the abundant availability of visible light in sunlight. Photocatalysis, a type of advanced oxidation process (AOP), is a chemical change that occurs when a catalytic material is activated due to its capacity to trap sunlight energy.^110^ This process is clean and has the potency of mineralizing EDCs into mineral compounds that benefit the environment. This process utilizes oxidation mechanisms, involving hydrogen peroxide-based degradation to break down the contaminants. Photocatalysts are semiconductor materials with a small energy bandgap, making them suitable for photocatalysis. Firstly, there is the generation of hydroxyl radicals in water, followed by a reaction between the hydroxyl radical and the micropollutants, resulting in the complete mineralization of the EDCs. Heterogeneous photocatalysis is a predominant prototype of photocatalysis in which the catalyst is in a different phase than the reaction medium. It uses light-absorbing solid material to generate both redox and oxidizing species in the presence of ultraviolet or visible light spectrum.^111^ Some of the commonly used heterogeneous photocatalysts include titanium dioxide (TiO_2_), zinc oxide (ZnO), bismuth ferrite (BiFeO_3_), and ferrite oxide (FeO_3_). However, in application, ZnO exhibit better EDC removal efficiency compared to TiO_2._ Effective photocatalytic degradation requires irradiation, typically with ultraviolet (UV) light, to activate the formation of electron–hole pairs and generate reactive charge carriers. These carriers produce hydroxyl radicals at the catalyst surface, driving the degradation of EDCs. Notably, removal efficiencies of 99% and 99.7% for bisphenol A (BPA) have been achieved using polyaniline-supported Ag@TiO_2_ nanocomposites under visible and UV light, and polyaniline-wrapped TiO_2_ nanorods under UV light, respectively. These high degradation rates are attributed to the combined activity of photogenerated holes (h^+^), hydroxyl radicals (˙OH), and superoxide radicals (˙O_2_^−^). Moreover, the incorporation of nitrate (NO_3_^−^) in polyaniline has been shown to further enhance BPA degradation. This is due to nitrate photolysis, which generates additional ˙OH radicals, thereby accelerating the mineralization process.^112^

Biodegradation is another low-cost and sustainable solution that is currently being utilized in the treatment of EDCs in groundwater through the activities of microorganisms and enzymes. The use of fungal reactor, bacteria, and activated sludge materials can successfully degrade EDCs like bisphenol A in groundwater.^4,113^ In a laboratory simulated study, white-rot fungi such as *Phanerochaetes ordida*, *Trametes versicolor*, *Pleurotus ostreatus*, *Aspergillus* spp. degraded BPA by 80–100% under ligninolytic and non-ligninolytic conditions.^114,115^ Additionally, a laccase enzyme cocktail derived from *Pycnoporus sanguineus* successfully removed 89–100% of BPA, 4-nonylphenol, 17α-ethynylestradiol, and triclosan from real groundwater samples, achieving 55–93% removal of these contaminants *in situ* the, although not from the shores of Africa.^116^ Similarly, the use of bacterial strains like *Pseudomonas*, *Acinetobacter*, *Sphingomonas* effectively degraded BPA over a period by making BPA its sole source of carbon.^117^ The use of hybrid constructed wetlands has been used to reduce the presence of heavy metals in some sub-Saharan groundwater samples to the WHO permissible limit.^118^ Nonetheless, adsorption and photocatalysis seem to be largely deployed for the removal of EDCs in African groundwater. Table 4 highlights selected studies on the use of adsorption and photocatalysis for the treatment of endocrine-disrupting compounds (EDCs) in groundwater systems.

**Table 4:** Various types of photocatalyst/adsorbents and targeted EDCs

Photocatalyst/adsorbent	EDCs	Degradation/adsorption efficiency	Notable findings	Reference
ZnO-biochar/kaolinite/chitosan/graphene oxide	17β-Estradiol (E2), 17α-ethynyl estradiol (EE2) and triclosan (TCS)	100% for 5 mg. L^−1^ E2, EE2 after 60 min and 10 mg L^−1^ 97.8% after 120 min for TCS	Superoxide radical from the synergistic effects of the materials played a major role in the degradation of the contaminants in tap water with four cycles efficiency without any decrease	119
Kaolinite clay, Na_2_WO_4_, titania, and plantain peel (biomass)	Ampicillin, sulfamethoxazole, and artemether	>90% photodegradation efficiency within 30 min for the targeted EDCs	Incorporation of the biomass enhanced the photocatalytic activity of the nano-composite and mineralized these contaminants to byproducts below the WHO permissible limit	120
Kaolinite clay-tungstates of Cu, Fe, and Zn, and *Carica papaya* seeds (pawpaw seed)	Acetaminophen, ampicillin and sulfamethoxazole	Of the three tungstate metals, Cu–ZnWO_4_–kaolin gave 100% degradation efficiency for ampicillin, 83% for acetaminophen, and 68% for sulfamethoxazole	The composite was still efficient even after five cycles, with about 90% removal efficiency for ampicillin. Generated by-products were within the WHO drinking water permissible limit	121
ZnWO_4_-kaolinite	Ampicillin	98% removal efficiency	The materials still had up to 90% degradation efficiency after five cycles	122
Ceramic filters and solar disinfection doped on TiO_2_	14 pharmaceuticals, 5 personal care products, and 9 pesticides	The two techniques had up to 90% removal efficiency for most of the targeted EDCs	For very recalcitrant compounds such as sulfadoxin, the material was improved upon by introducing a photocatalyst (TiO_2_)	91
p–n ZnO/GO	Estrone (E1), 17-β-estradiol (E2), estriol (E3) and the synthetic estrogen 17-α ethinylestradiol (EE2)	Estrogen removal was >89% and as high as 98%	The photocatalyst was efficient in both the single and combined solute mixture, and it was still efficient after 3 cycles	123
CZPPrGO	Ibuprofen (IBP) and diclofenac (DCF)	>80% removal for both IBP and DCF	The adsorbent was still efficient after 4 cycles	124
La_0.8_FO and La_0.8_FO@PgNS	Sulfamethoxazole (SMX)	52.06 and 99.60% degradation efficiency for SMX	La_0.8_FO@PgNS was stable over 8 cycles	125
Citric acid-functionalized mesoporous MCM-41 (C-MCM-41)	Ciprofloxacin (CIP)	>90% CIP degradation	The adsorbent is still efficient after 4 reuse cycles	119
NiFe@BBAL	Ciprofloxacin (CIP) and metronidazole (MET)	>96% removal for CIP and MET	The adsorbent proved to be efficient after 5 reuse cycle and it was not toxic	126

## 6. Policy recommendation for EDCs management in African groundwater

To effectively address the growing threat of EDCs pollution in African groundwater sources, targeted policy recommendations and interventions are urgently needed. There is a need to establish national and regional EDC-specific regulations and water quality standards.^12^ Additionally, the continent should build a robust monitoring system. Methodologies should be developed that can monitor EDCs *in situ* as recommended by the OECD. The Government and private establishments should strengthen the laboratories and invest in technical and capital building so that multiple-residual analysis of EDCs can be carried out, as seen in South Africa's groundwater system. Furthermore, EDC surveillance should be incorporated within existing national and regional groundwater governance and aquifer-protection zoning, leveraging on the African Ministers' Council on water for regional harmonization and collaborations.^127^ More importantly, sensitization should be heightened specifically, in underserved settings, on proper EDCs disposal, waste management, and management of groundwater systems *via* formal and informal methods.^128^ This multi-disciplinary approach aims to build a resilient and structured regulatory system that will enhance early detection and safeguard the groundwater systems across the continent of Africa.

## 7. Challenges, future direction, and stakeholder collaboration

The high cost of sampling and instrumentation techniques is highly responsible for the deficit of data in most parts of Africa. This has largely led to the continent's inability to develop robust and well-tailored exposure models and risk assessment frameworks. As a result, there is a need for more local and regional studies on the continents, as the usage of EDCs may differ across countries. Data generated across different regions will be imperative in the development and implementation of robust problem formulation, development of conceptual exposure models, and risk assessment frameworks for the continent. Furthermore, the structural complexity and diverse modes of EDCs mechanisms present significant challenges that demand sensitive multi-class analytical methods that are capable of detecting a broad spectrum of EDCs in groundwater sources and other aquatic matrices. As future directions, the continent must prioritize the development of low-cost remediation systems, expand analytical capacity, and coordinate surveillance initiatives that are informed, inclusive, and sustainable, thereby safeguarding groundwater resources and public health. More importantly, sensitization and advocacy on the abuse of some of these chemicals should be increased, especially in low-income areas where some of them engage in unregulated local production. It is worth noting that addressing these challenges is dependent on the active participation of all relevant stakeholders. Significant regional bodies like AMCOW and the Water and Sanitation Sector Monitoring and Reporting System (WASSMO) should collaborate with academic institutions to develop efficient, effective, and low-cost analytical techniques for the environmental surveillance of EDCs in the continent's predominant drinking water supply. Also, the integration of policy-makers and civil society can ensure that research findings are translated into actionable insights and community-level interventions, where necessary.

## 8. Conclusion

Our analysis of the provided dataset demonstrates that African groundwater is contaminated with a broad array of EDCs. Many detected concentrations exceed WHO or US EPA drinking-water values by factors of 10 to 100, particularly for pesticides. The review validates a lack of comprehensive region-specific studies that identify the temporal and spatial distribution of EDCs as well as prevalent anthropogenic channels that contribute to the co-occurrence of EDCs in groundwater sources. Also, there is insufficient understanding of the eco-human toxicity of EDCs in groundwater. These research gaps across the region are largely driven by weak monitoring and analytical techniques. Additionally, poor policy formulation and regulatory framework, as well as low public awareness, attenuate the increased concentration observed in the few studies conducted in the region. Given that EDCs contamination in groundwater poses significant risks to environmental health and socio-economic stability, its protection is paramount. While research has predominantly focused on EDC contamination in surface water due to its direct exposure to wastewater effluents and sludge, the threat to groundwater remains a pressing concern. To address this alarming research, there is an urgent need for continuous region-specific monitoring of EDCs to identify temporal and spatial trends, as well as the prevalent anthropogenic channels. Hydrogeological modelling should be emphasized for a better understanding of environmental and exposure pathways of EDCs in groundwater. Policy formulation and implementation of groundwater quality standards should be strengthened by integrating prevalent EDCs in each region. The continent should develop regional monitoring and data-sharing frameworks, invest in public education, encourage sustainable agricultural practices, regulate the use of preservatives, especially in the informal sector, and support community-based participatory research. Typically, addressing this pertinent issue requires a comprehensive and multidisciplinary approach that integrates scientific research, policy enforcement, technological advancements, and community engagement. Implementing sustainable solutions is essential to preserving water resources and mitigating the harmful effects of EDC exposure on human and ecological health.

## Author contributions

Esther A. Nnamani: data curation, formal analysis, investigation, methodology, software, resources, validation, visualization, writing–original draft, writing-review and editing; Ajibola A. Bayode: data curation, formal analysis, investigation, methodology, software, resources, validation, visualization, writing–original draft, writing–review and editing; Oluwaferanmi B. Otitoju: formal analysis, investigation, software, resources, validation, writing–original draft; Moses O. Alfred: formal analysis, methodology, visualization, writing–review and editing; Martins O. Omorogie: conceptualization, data curation, formal analysis, funding acquisition, investigation, methodology, project administration, software, resources, supervision, validation, visualization, writing–original draft, writing–review and editing.

## Conflicts of interest

The authors declare that they have no known competing financial interests or personal relationships that could have appeared to influence the work reported in this paper.

## Data Availability

No data was used for the research described in this paper.
